# Promising clinical outcomes of sequential and “Sandwich” chemotherapy and extended involved‐field intensity‐modulated radiotherapy in patients with stage I_E_/II_E_ extranodal natural killer/T‐cell lymphoma

**DOI:** 10.1002/cam4.1755

**Published:** 2018-11-28

**Authors:** Han‐yu Wang, Shao‐qing Niu, Yun‐ying Yang, Yi‐yang Li, Hong‐bo Chen, Yu‐jing Zhang

**Affiliations:** ^1^ Department of Radiation Oncology State Key Laboratory of Oncology in South China Collaborative Innovation Sun Yat‐sen University Cancer Center Guangzhou Guangdong China; ^2^ Department of Radiation Oncology The First Affiliated Hospital of Sun Yat‐sen University Guangzhou Guangdong China; ^3^ Department of Oncology The First Affiliated Hospital of Guangdong Pharmaceutical University Guangzhou Guangdong China

**Keywords:** extended involved‐field intensity‐modulated radiotherapy, extranodal natural killer/T‐cell lymphoma, loco‐regional recurrence, nasal‐type, prognosis, sequential chemoradiotherapy

## Abstract

**Background:**

The optimal treatment for the rare subtype of non‐Hodgkin lymphoma, extranodal natural killer/T‐cell lymphoma (ENKTL), nasal‐type, has not been clearly defined. The purpose of the study was to investigate the efficacy of sequential and “Sandwich” chemotherapy and extended involved‐field intensity‐modulated radiotherapy (IMRT) in patients with stage I_E_/II_E_ extranodal ENKTL, nasal‐type.

**Methods:**

One hundred and fifty‐five patients with stage I_E_/II_E_ nasal‐type ENKTL were enrolled in the study, including 99 patients treated with sequential chemotherapy and extended involved‐field IMRT (SCRT) and 56 patients with “Sandwich” chemotherapy and extended involved‐field IMRT and chemotherapy (SCRCT). All patients were treated with extended involved‐field IMRT with median dose of 54.6 Gy to the primary tumor and positive lymph nodes. Ninety‐four patients had Ann Arbor stage I_E_ disease, and 61 patients had stage II_E_ disease.

**Results:**

The 5‐year rates of loco‐regional recurrence (LRR), progression‐free survival (PFS), and overall survival (OS) were 17.0%, 78.5%, and 84.7%, respectively. Univariate analysis revealed that EBV DNA copy after treatment (normal vs elevated level) was significant prognostic factor for LRR, PFS, and OS (*P < *0.001); therapeutic method (SCRT vs SCRCT) was significant prognostic factor for PFS (71.0% vs 91.8%, *P *= 0.011), but there was no significant effect on 5‐year LRR and OS (22.2% vs 8.2%, *P *= 0.051 for LRR; 80.9% vs 91.8%, *P *= 0.199 for OS).

**Conclusions:**

Compared with SCRT, SCRCT was significantly associated with higher PFS rates and showed a trend toward improved loco‐regional control. EBV DNA copy after treatment is a good index for recurrence and prognosis for stage I_E_/II_E_ ENKTL patients.

## 1 INTRODUCTION

Extranodal natural killer/T‐cell lymphoma (ENKTL), nasal‐type, is a distinct subtype of non‐Hodgkin lymphoma (NHL) that is common in Asia but rare in Europe and North America.^1^, ^2^, ^3^, ^4^, ^5^, ^6^ The upper aerodigestive tract is the most commonly involved site,^3^, ^7^ particularly the nasal cavity and Waldeyer's ring. Because of the rarity of ENKTL worldwide and its heterogeneity, optimal treatment strategies have not been defined to date.

Previous studies have shown that ENKTL is sensitive to radiotherapy,^8^, ^9^, ^10^, ^11^, ^12^, ^13^, ^14^, ^15^ but resistant to conventional chemotherapy due to the overexpression of the multidrug‐resistant P‐glycoprotein.^16^, ^17^ After treatment with concurrent chemoradiotherapy (CCRT), Kim et al^18^ reported 3‐year progression‐free survival (PFS) and overall survival (OS) as high as 85.19% and 86.28%, respectively, in patients with stage I_E_ /II_E_. Another prospective research study,^19^ which adopted CCRT, reported a 5‐year OS of 70% for localized nasal natural killer/T‐cell lymphoma. However, the previous two studies adopted three‐dimensional conformal radiotherapy (3D‐CRT); currently, with the rapid development of radiotherapy technology, IMRT has been widely applied in clinical work as it is more precise and provides better dose coverage.^20^, ^21^ This aim of this study was to explore the clinical outcomes in early‐stage ENKTL patients treated with sequential (chemotherapy‐IMRT, SCRT) or “Sandwich” (chemotherapy‐IMRT‐chemotherapy, SCRCT) chemoradiotherapy.

## 2 MATERIALS AND METHODS

### 2.1 Patient eligibility criteria

One hundred and fifty‐five patients with stage I_E_ or II_E_ ENKTL who received sequential (SCRT) or “Sandwich” (SCRCT) chemotherapy and extended involved‐field IMRT consecutively in Sun Yat‐Sen University Cancer Center between January 2010 and August 2015 were recruited in this study. The sites of primary tumor were located within the nasal cavity (n* *= 120) or Waldeyer's ring (n* *= 32), and the other sites of upper aerodigestive tract (n* *= 3). The diagnostic criteria were based on the 2008 WHO classification of Tumours of Haematopoietic and Lymphoid Tissues, and every case was diagnosed after a consensus was reached among at least two experts.

The majority of patients who showed the pathologic features of angiocentricity zone necrosis and polymorphism of individual cells and tumor cells also expressed NK/T‐cell markers, such as CD2,CD3ε(+), cytotoxic granule proteins (TIA‐1, granzyme‐B, and perforin), CD56, and EBV encoded small RNA in situ hybridization, but they did not express B‐cell markers such as CD20 and/or CD79α.

Patients were staged according to the Ann Arbor staging system. Clinical evaluation of patients included history and physical examination, contrast‐enhanced magnetic resonance imaging (MRI) of the head and neck, complete blood count, liver and renal function tests, serum lactate dehydrogenase levels (LDH), contrast‐enhanced CT of the chest and abdomen/pelvis, bone marrow aspiration and/or biopsy. Positron emission tomography/computed tomography (PET/CT) was performed in 97 patients.

### 2.2 Treatment

Ninety‐nine patients received sequential chemotherapy and extended involved‐field IMRT (SCRT) and 56 patients received “Sandwich” chemotherapy followed by extended involved‐field IMRT and chemotherapy (SCRCT).

RT was delivered using extended involved‐field IMRT with 6‐MV photon beams, and all plans were calculated using the Eclipse or Monaco system. The median radiation doses were 54.6 Gy (46.0‐60.9 Gy) for primary tumor or positive lymph nodes, 50.7 Gy (46.0‐56.0 Gy) for high‐risk clinical target volume (CTV1), and 45.5 Gy (36.0‐52 Gy) for low‐risk clinical target volume (CTV2). Gross tumor volume (GTV) was defined as the gross tumor extent shown on the imaging studies and physical examination before treatment, including the primary tumor and involved regional lymph nodes. High‐risk clinical target volume (CTV1) included GTV and adjacent structures in risk, such as nasal mucosa, nasopharyngeal mucosa, the retropharyngeal lymph nodes, the Waldeyer's ring, and ethmoid sinus. The low‐risk clinical target volume (CTV2) included the corresponding neck lymphatic drainage area, such as the upper cervical lymph node was included when nasopharynx or retropharyngeal lymph node was involved and the whole cervical lymph node was included when an upper cervical lymph node was involved.

### 2.3 Chemotherapy

Eighty‐five patients (54.8%) were treated with GELOX (gemcitabine, oxaliplatin, and L‐asparaginase/pegaspargase) or GELOX‐like regimen (median: four cycles; range, 1‐6 cycles); 20 patients (12.9%) received CHOP (cyclophosphamide, doxorubicin, vincristine, and prednisolone) or CHOP‐like regimen (median: five cycles; range, 2‐6 cycles), 19 patients (12.2%) received GAD‐M regimen (gemcitabine, l‐asparaginase/pegaspargase, methotrexate, dexamethasone) (median: six cycles; range, 2‐6 cycles), 11 patients received ATT alternative regimen (DHAP [cisplatin, high‐dose cytarabine, dexamethasone], CHOPB [cyclophosphamide, vincristine, THP‐doxorubicin, bleomycin, prednisone], and IMVP16 [ifosfamide, methotrexate, etoposide, dexamethasone] alternately; median: five cycles; range, 2‐6 cycles), four patients received VIPD regimen (etoposide, ifosfamide, cisplatin, and dexamethasone), and one patient received DeVIC (dexamethasone, etoposide, ifosfamide, and carboplatin) regimen. Fifteen patients received other regimens. In SCRCT group, the median chemotherapy cycle before and after IMRT was both three cycles (range 1‐6). Chemotherapy was repeated every 3 weeks.

### 2.4 Statistical analysis

The primary endpoint was loco‐regional recurrence (LRR), and the secondary endpoints were progression‐free survival (PFS) and overall survival (OS). The response was evaluated based on the International Workshop Criteria reported in 1999.^22^ LRR was defined as a relapse at the primary tumor site, adjacent organ/structure, or regional lymphoma nodes. Complete remission (CR) was defined as a complete regression of all visible/palpable tumors and radiographic disease. OS was measured from the start of initial treatment until time of death from any causes or until last follow‐up. PFS was measured from the start of initial treatment until time of first local or distant progression or relapse, or until last follow‐up, or death. A survival curve was constructed using the Kaplan‐Meier method, and the groups were compared using the log‐rank test. A *P* value < 0.05 was considered statistically significant. All statistical analyses were performed using IBM SPSS Statistics, version 22.0 (IBM Corp., Armonk, NY, USA).

## 3 RESULTS

### 3.1 Patient characteristics

The clinical features of all 155 patients are summarized in Table 1. The ratio of men to women was 2.03:1. The median age was 42 years (range, 13‐75), 35 patients (22.6%) had elevated LDH, and 72 patients (46.5%) presented “B” symptoms. According to KPI (Korean Prognostic Index), there were 102 patients with 0‐1 score, 53 patients with more than 2 score. Among the 133 patients whose EBV DNA copies were documented before treatment, there were 71 patients (53.4%) with elevated copies.

**Table 1:** Clinical characteristics and univariate analysis of prognostic factors for 155 patients

Prognostic factor	No.	5‐y LRR		5‐y PFS		5‐y OS	
		%	*P*	%	*P*	%	*P*
Age, years							
≤60	145	16.3	0.664	80.0	0.360	87.0	0.513
60	10	28.0		60.0		64.3	
Ann Arbor stage							
I	94	13.6	0.135	77.7	0.607	85.7	0.278
II	61	22.5		79.0		83.7	
“B” symptom							
No	83	15.9	0.523	74.9	0.438	81.4	0.573
Yes	72	18.3		83.1		89.2	
Serum LDH							
Normal	114	16.3	0.747	83.3	0.536	84.4	0.788
Elevated	35	18.5		79.9		87.3	
NA	6	16.7		74.5		83.3	
EB DNA copy after treatment							
Normal	119	12.5	<0.001	83.0	<0.001	90.0	<0.001
Elevated	16	58.3		30.0		41.7	
NA	20	11.9		88.4		88.1	
Time from diagnosis to radiotherapy							
≤3 mo	86	12.7	0.095	83.0	0.151	84.7	0.114
3 mo	66	22.1		72.8		86.4	
NA	3	33.3		66.7		66.7	
Involvement of adjacent structure							
No	44	14.9	0.702	83.5	0.317	87.5	0.215
Yes	111	18.1		76.5		83.6	
Therapeutic method							
SCRT	99	22.2	0.051	71.0	0.011	80.9	0.199
SCRCT	56	8.2		91.8		91.8	
KPI							
0‐1	102	16.3	0.768	76.9	0.615	82.9	0.662
≥2	53	18.4		81.7		88.8	
Response after treatment							
CR	124	15.3	0.137	79.8	0.210	87.2	0.019
Non‐CR	20	20.7		75.0		79.3	
NA	11	29.9		72.7		69.3	

EBV, Epstein‐Barr virus; ECOG, Eastern Cooperative Oncology Group; KPI, Korea Prognostic Index; LDH, lactate dehydrogenase; NA, not available.

### 3.2 Treatment response

When all patients completed chemotherapy and extended involved‐field IMRT, 144 patients were assessable for a response, including 124 cases (86.1%) who achieved CR and 16 cases who achieved PR, one patient had SD and three patients had PD.

### 3.3 Survival and prognostic factors

**Figure 1:**
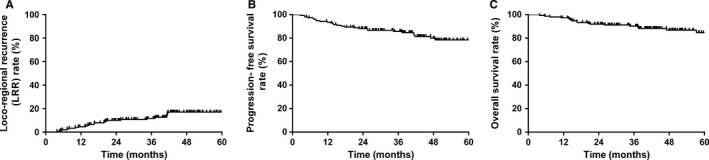
Kaplan‐Meier survival curves for all patients in this study. The 5‐y loco‐regional recurrence (LRR) rate for all patients is 17.0% (A). The 5‐y progression‐free survival (PFS) rate for all patients is 78.5% (B). The 5‐y overall survival (OS) rate for all patients is 84.7% (C)

The median follow‐up time for survival was 44.5 months (range 3.8‐84.1 months). The 5‐year LRR, PFS, and OS rates for all patients were 17.0%, 78.5%, and 84.7%, respectively (Figure 1).

Patients’ characteristics were evaluated for prognostic significance against LRR, PFS, and OS (Table 1). According to univariate analysis results, the following variables were associated with the 5‐year OS rate: EBV DNA copy after treatment (normal vs elevated level, *P < *0.001) and response after treatment (CR vs no‐CR, *P *= 0.019). Elevated EBV DNA copy after treatment (normal vs elevated level, *P < *0.001) and therapeutic method (SCRT vs SCRCT, *P *= 0.011) were found to be significant prognostic factors for 5‐year PFS. Elevated EBV DNA copy after treatment (*P < *0.001) was found to be significant prognostic factors for 5‐year LRR, and therapeutic method (SCRT vs SCRCT, *P *= 0.051) showed a trend toward improved loco‐regional control (Table 2).

**Table 2:** Multivariate analysis of 155 patients with I‐II stage ENKTCL

Variable	Overall survival			Progression‐free survival		
	HR	95% CI	*P*	HR	95% CI	*P*
“B” symptom (yes vs no)	0.743	0.160‐3.457	0.705	1.561	0.419‐5.817	0.507
Ann Arbor stage (I vs II)	1.044	0.256‐4.252	0.952	1.653	0.476‐5.738	0.429
Serum LDH (normal vs elevated)	0.712	0.153‐3.324	0.666	1.423	0.392‐5.169	0.592
EBV DNA copy after treatment (normal vs elevated level)	1.609	0.724‐3.575	0.243	1.308	0.806‐2.123	0.278
Response after treatment (CR vs no‐CR)	2.128	0.976‐4.639	0.057	1.474	0.712‐3.051	0.296
Time from diagnosis to radiotherapy (≤3 vs >3 mo)	1.443	0.554‐3.759	0.453	1.511	0.647‐3.530	0.340

**Figure 2:**
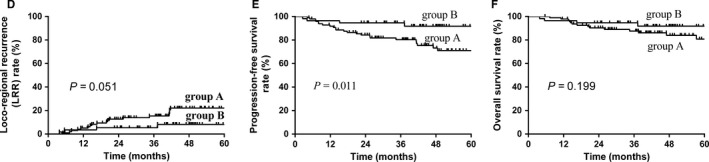
The comparison of loco‐regional recurrence (LRR), progression‐free survival (PFS), and overall survival (OS) rates between patients in two groups with two different treatment modes (Group A: 99 patients who were treated with sequential chemotherapy and extended involved‐field IMRT [SCRT]; Group B: 56 patients who were treated with “Sandwich” chemotherapy and extended involved‐field IMRT and chemotherapy [SCRCT]). The 5‐y LRR of patients in group A and group B are 22.2% vs 8.2% (*P *= 0.051), respectively (D). The 5‐y PFS of patients in group A and group B is 71.0% vs 91.8%, respectively (*P *= 0.011) (E). The 5‐y OS of patients in group A and group B are 80.9% vs 91.8%, respectively (*P *= 0.199) (F)

According to subgroup analysis, there are significant differences in PFS between SCRT group and SCRCT group. The 5‐year LRR, PFS, and OS rates were 22.2%, 71.0%, and 80.9% for SCRT group, respectively. For patients treated with SCRCT, the 5‐year LRR, PFS, and OS rates were 8.2%, 91.8%, and 91.8% (*P *= 0.051 for LRR, *P *= 0.011 for PFS, *P *= 0.199 for OS; Figure 2).

### 3.4 Toxicities

Data on toxicities are summarized in Table 3. No patient developed Grade 3 or 4 toxicity. The most frequent toxicity was mucositis. In SCRT group, mucositis was scored as Grade 1 in 51 patients (51.5%) and Grade 2 in 19 patients (19.2%). In SCRCT group, mucositis was scored as Grade 1 in 32 patients (57.1%) and Grade 2 in 12 patients (21.4%).

**Table 3:** Incidence of toxicities in patients

Acute toxicities	Grade, n (%)					
	0		1		2	
	SCRT	SCRCT	SCRT	SCRCT	SCRT	SCRCT
Mucositis	29 (29.3)	12 (21.4)	51 (51.5)	32 (57.1)	19 (19.2)	12 (21.4)
Xerostomia	43 (43.4)	22 (39.3)	37 (37.4)	24 (42.9)	19 (19.2)	10 (17.8)
Dysphagia	50 (50.5)	26 (46.4)	40 (40.4)	24 (42.9)	9 (9.1)	6 (10.7)
Fever	91 (91.9)	50 (89.3)	7 (7.1)	5 (8.9)	1 (1.0)	1 (1.8)
Leukopenia	74 (74.7)	38 (67.8)	25 (25.3)	16 (28.6)	—	2 (3.6)
Anemia	82 (82.8)	45 (80.4)	17 (17.2)	11 (19.6)	—	—
Thrombocytopenia	94 (94.9)	50 (89.3)	5 (5.1)	6 (10.7)	—	—

SCRT, sequential chemotherapy and IMRT; SCRCT, chemotherapy‐IMRT‐chemotherapy.

The second most frequent toxicity was xerostomia. In SCRT group, Grade 1 and 2 xerostomia were detected in 37 (37.4%) and 19 (19.2%) patients, respectively. In SCRCT group, Grade 1 and 2 xerostomia were detected in 24 (42.9%) and 10 (17.9%) patients, respectively.

## 4 DISCUSSION

The main finding of this study is that, compared with SCRT, SCRCT was significantly associated with higher PFS rates and showed a trend toward improved loco‐regional control (PFS: 71.0% vs 91.8%, *P *= 0.011; LRR: 22.2% vs 8.2%, *P *= 0.051). Another valuable finding is that EBV DNA copy after treatment is significantly associated with 5‐year LRR, PFS, and OS.

In order to improve the local and systemic disease control for ENKTL patients, Yamaguchi et al^19^, ^20^, ^21^, ^22^, ^23^ performed the first prospective trial of CCRT involving 27 patients with localized nasal NKTCL treated with concurrent 3D‐CRT (50 Gy) and three cycles of DeVIC. They showed a 5‐year OS of 70% and PFS of 63%. Kim et al^18^ enrolled 30 stage I_E_/II_E_ nasal ENKTL patients who received CCRT (3D‐CRT radiation, 40‐52.8 Gy and cisplatin, 30 mg/m^2^ weekly) followed by three cycles of VIPD resulting in 3‐year PFS and OS rates of 85.19% and 86.28%, respectively. The two studies achieved satisfactory outcomes by CCRT; however, in the two studies, 3D‐CRT radiation was adopted as RT. In contrast, IMRT is now widely used in clinical practice as it can achieve superior target coverage and dose conformity compared with 3D‐CRT and provides equivalent or slightly better organs at risk (OARs) avoidance compared with 3D‐CRT.^20^, ^21^

This study also achieved excellent LRC and favorable prognoses for patients with stage I_E_/II_E_ ENKTL compared with the two CCRT studies.^20^, ^21^ Our favorable clinical results are likely due to IMRT adopted in all patients. IMRT was been proven to achieve excellent target coverage and dose conformity, as well as favorable prognosis and LRC rates with acceptable toxicities in patients with nasal and Waldeyer's ring NKTCL (WR‐NKTCL).^24^, ^25^ Wang et al^25^ analyzed 42 patients with early‐stage nasal NK/T‐cell lymphoma who received high‐dose (ie, median radiation dose to the primary tumor of 50 Gy) and extended involved‐field IMRT with or without combination chemotherapy and reported 2‐year LRC, OS, and PFS rates of 93%, 78%, and 74%, respectively. Similarly, Bi et al^24^ retrospectively reviewed 30 patients with early‐stage WR‐NKTCL who received high‐dose (ie, 50 Gy to the primary involved regions and positive cervical lymph nodes and 40 Gy to the negative cervical nodes) and extended‐field IMRT and reported 2‐year OS, PFS, and LRC rates of 71.2%, 57.4%, and 87.8%, respectively.

Another possible reason for our success may be related to the fact that the majority of patients in this study received GELOX or GELOX‐like chemotherapy regimens, which is not affected by P‐glycoprotein and produces a better prognosis with less toxicity than EPOCH /CHOP in early‐stage ENKTL patients.^26^, ^27^, ^28^ Previous studies have shown that CHOP or CHOP‐like regimens lead to inferior treatment outcomes. Wang et al^28^ ^28^ reported the 2‐year OS and PFS were both 86% for stage I_E_/II_E_ ENKTL patients treated with GELOX followed by involved‐field radiation.

In this study, mucositis and xerostomia are the most common radiotherapy‐related toxicities, but no patient developed Grade 3 or 4 toxicities were documented. The reason for the mild radiotherapy‐related toxicities is that all patients were treated with IMRT, which had been proven to well protect OARs, such as parotid gland. Compared with SCRT group, patients in SCRCT group were more frequently to develop hematologic toxicities, such as leukopenia, anemia, and thrombocytopenia, and this may be the results of the more cycles of chemotherapy in SCRCT group.

In conclusion, sequential and “Sandwich” chemotherapy (GELOX‐based) combined with extended involved‐field IMRT could get ideal clinical outcome. Compared with SCRT, SCRCT could get higher PFS rates and show a trend toward improved loco‐regional control. EBV DNA copy after treatment is a good index for recurrence and prognosis for stage I_E_/II_E_ ENKTL patients.

## CONFLICT OF INTEREST

Nothing to report.
